# A Review of Black Salve: Cancer Specificity, Cure, and Cosmesis

**DOI:** 10.1155/2017/9184034

**Published:** 2017-01-26

**Authors:** Andrew Croaker, Graham J. King, John H. Pyne, Shailendra Anoopkumar-Dukie, Lei Liu

**Affiliations:** ^1^Southern Cross Plant Science, Southern Cross University, Lismore, NSW 2480, Australia; ^2^Wesley Medical Research Institute, Wesley Hospital, Auchenflower, QLD 4066, Australia; ^3^School of Medicine, University of Queensland, St Lucia, QLD 4072, Australia; ^4^School of Pharmacy, Griffith University, Gold Coast Campus, Gold Coast, QLD 4222, Australia

## Abstract

Black salve is a topical escharotic used for the treatment of skin cancer. Although promoted as a safe and effective alternative to conventional management by its proponents, limited clinical research has been undertaken to assess its efficacy and potential toxicities. Patients are increasingly utilizing the Internet as a source of health information. As a minimally regulated space, the quality and accuracy of this information vary considerably. This review explores four health claims made by black salve vendors, investigating its natural therapy credentials, tumour specificity, and equivalence to orthodox medicine in relation to skin cancer cure rates and cosmesis. Based upon an analysis of in vitro constituent cytotoxicity, in vivo post black salve histology, and experience with Mohs paste, black salve is likely to possess normal tissue toxicity with some cancer cell lines being relatively resistant to its effects. This may explain the incongruous case study reports of excessive scarring, deformity, and treatment failure.

## 1. Introduction

Complementary and alternative medicines (CAM) encompass a wide range of popular health interventions [1]. Self-reported use of CAM from surveys across North America, Europe, and Australia shows a high level of use with 35 to 69% of respondents having used CAM in the preceeding 12 months [2–4].

Patients with dermatological conditions more heavily utilize CAM than the general population, with 12-month prevalence figures of 49% versus 36% in 2002 [5] and 85% versus 38% in 2007 [6]. Despite conventional therapies having high cure rates for skin malignancies, a number of patients are opting to manage their skin cancers with CAM [7]. While the extent of this practice is yet to be determined, patients are motivated by a preference for natural therapies and a perception of lower toxicity for CAM compared to orthodox treatment modalities [8].

Despite questions regarding the effectiveness of CAM therapies [9] and their general lack of systematic scientific testing [10], herbal medicines should not be dismissed without consideration. Natural products have been a vital source of anticancer pharmaceuticals, with 49% of oncology small molecules discovered since 1940 being either unaltered or direct derivatives of natural compounds [11]. Due to the molecular diversity and structural complexity of natural products compared to synthetic compound libraries, there is renewed interest in natural product drug discovery [12].

Concerns however exist about the lack of regulatory control over the natural therapy sector and how this impacts quality control and patient safety [13]. Although used as medicines, regulators in some countries treat herbal therapies as dietary supplements [14]. Unlike pharmaceutical products, manufacturers are not always required to provide safety or efficacy evidence to regulators before their product can be sold to patients [15]. To better inform clinicians and patients, this literature review seeks to assess some of the health claims made regarding black salve.

## 2. Black Salve as a Natural Therapy

Black salve was originally developed by an American surgeon, Jesse Fell, its use first reported in the 1850s [16]. Fell had heard of a plant growing on the shores of Lake Superior used by Native Americans to treat cancer (reviewed in [17]). He identified it as* S. canadensis*, combining it with zinc chloride to make a cancer salve known as Fells' paste [18]. Since then, other entrepreneurs have developed topical cancer therapies based on these two core ingredients, today's formulations being known as black salve [19].

Referred to as “Natures Scalpel” [20], it is important to assess the veracity of black salves' natural credentials. Consumers often believe natural therapies are safe, having the ability to heal with a low risk of toxicity [21]. Portraying black salve as a natural product appeals to this naturalness bias, which in turn may increase black salve utilization by CAM users.

Black salves usually contain bloodroot, the rhizome of* Sanguinaria canadensis*. Manufacturers have differing formulations that may also contain chaparral* (Larrea mexicana)*, graviola* (Annona muricata*), oleander* (Nerium oleander)*, galangal* (Alpinia officinarum)*, ginger* (Zingiber officinale)*, red clover* (Trifolium pratense)*, sheep sorrel* (Rumex acetosella)*, burdock* (Arctium lappa)*, pokeroot* (Phytolacca decandra),* and turmeric* (Curcuma longa)* [22–25].

Apart from its botanical ingredients, black salve contains zinc chloride (ZnCl_2_) with some formulations also containing dimethyl sulfoxide (DMSO). ZnCl_2 _is a chemical usually manufactured from zinc and hydrochloric acid and does not occur naturally apart from the very rare mineral simonkolleite Zn_5_(OH)_8_Cl_2_H_2_O [26]. ZnCl_2_ is widely used for industrial processes such as textile manufacture and metallurgical fluxes for soldering galvanized iron [27]. The ZnCl_2_ contained in black salve is a synthesized chemical and in some preparations the main ingredient by weight [28].

DMSO is added to enhance the epidermal penetration of some black salve formulations. Although trace amounts of DMSO may be naturally found in cereals, fruits, and vegetables [29], DMSO is commercially manufactured from lignin, a byproduct of paper production [30]. DMSOs' chemical synthesis utilizes lignins free methyl radicals; these are coupled to sulphur and then oxidized [31].

Patients seeking natural skin cancer therapies may not realize black salve contains significant quantities of synthetic chemicals. This knowledge may alter the treatment choices of CAM patients, a population often wanting to reduce their exposure to unnatural compounds [56].

## 3. Black Salve Cancer Specificity and Normal Tissue Toxicity

Black salve is promoted as a safe skin cancer therapy, able to discriminate between cancerous and healthy tissue [57]. There are concerns about the accuracy of this claim with cases of extensive tissue necrosis reported in the literature from black salve use [38, 58]. Two explanations may account for this finding. Treated cancers were more extensive than they appeared clinically, or, in addition to having a destructive effect on malignant tissue, black salve can also cause normal tissue necrosis.

As a therapeutic product containing multiple bioactive compounds, the discriminating ability of black salve can be gauged by the cytotoxic potential of its individual constituents against malignant and normal cells.* S. canadensis*, known as bloodroot because of the red latex of its rhizome, is an important component of black salve [17]. Native Americans called the plant puccoon and used it as a traditional medicine to treat a variety of conditions including cancer [18].

Bloodroot rhizomes predominantly contain a number of quaternary benzophenanthridine alkaloids (QBA) in addition to protopin alkaloids [59]. These plant defence toxins target multiple cellular pathways [60], being able to intercalate with DNA [61] and RNA [62–64], alter gene expression through the epigenetic modification of chromatin and core histones [65], inhibit microtubules needed for cell division [66], and generate oxidative stress [67].

Several studies have compared the antiproliferative and cytotoxic effects of these alkaloids against normal and malignant cells in vitro. In 2000, Ahmad et al. reported that sanguinarine had preferential, concentration dependent tumour cytotoxicity when tested against the A431 epidermoid carcinoma cell line and normal human epidermal keratinocytes derived from foreskin. At 2 *μ*M, sanguinarine induced 60% cytotoxicity in A431 cells compared to 10.8% in normal keratinocytes. However 5 *μ*M sanguinarine caused 65% cytotoxicity in A431 cells compared to 38.3% in normal keratinocytes representing a loss of discrimination [34].

**Figure 1:**
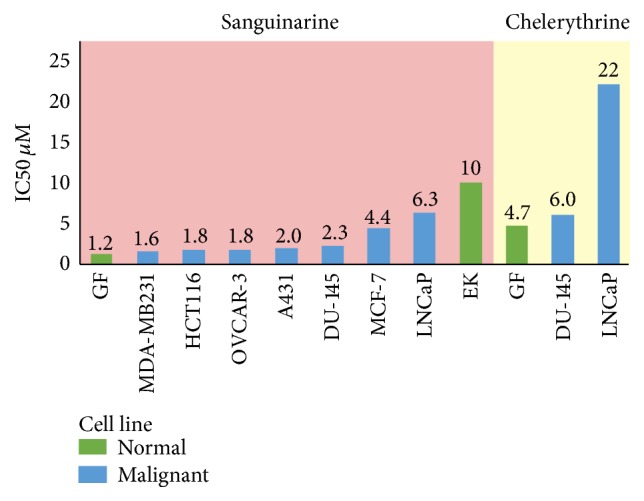
Major QBA cytotoxicity against normal and malignant cells 24 hr incubation. Data from [34, 69, 71]. Cell line: GF: gingival fibroblast; EK: epidermal keratinocyte; MDA-MB231: breast; HCT116: bowel; OVCAR-3: ovarian; A431: SCC; Du-145: prostate; MCF-7: breast; LNCaP: prostate.

**Figure 2:**
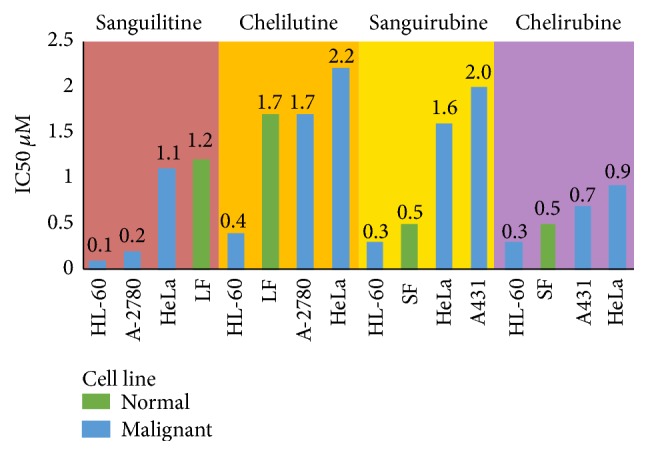
Minor QBA cytotoxicity against normal and malignant cells 72 hr incubation. Data from [32, 35]. Cell line: LF: lung fibroblast; SF: skin fibroblast; HL60: leukaemia; A-2780: ovarian; HeLa: cervical; A431: SCC.

Contradictory results however have shown sanguinarine to have greater cytotoxicity against normal skin fibroblasts compared to the same A431 squamous cancer cell line [32]. This suggests that sensitivity to sanguinarine cytotoxicity depends more on target cell characteristics than whether the exposed cells are malignant or benign. After 24-hour exposure to the two main bloodroot alkaloids sanguinarine and chelerythrine gingival fibroblasts have shown low IC_50_ values of 1.2 *μ*M and 4.7 *μ*M, respectively [33, 68] (Figure 1). Various cancer cell lines in these studies showed greater resistance to sanguinarine cytotoxicity than nonmalignant cells, further undermining the argument that sanguinarine has tumour specific cytotoxicity. Similar results from four biologically active* S. canadensis* minor quaternary benzophenanthridine alkaloids (minor QBAs) also frequently showed greater nonmalignant cell cytotoxicity (Figure 2).

The mechanism of sanguinarine cytotoxicity has not been fully elucidated and appears to vary for different cell types. In squamous carcinoma cells, sanguinarine induces cell death by apoptosis, while, in keratinocytes, sanguinarine induces cell death by necrosis [34]. Several apoptotic molecular pathways have been implicated such as caspase 3 and 7 activation in PC3 prostate cancer cells, rapid GSH depletion in murine L929 fibroblasts [68], and reduced bcl-2 with increased BH3 interacting domain death agonist (Bid) and proapoptotic B cell lymphoma 2 associated X protein (Bax) levels in gingival fibroblasts [69].

As epidermal keratinocytes provide a barrier to environmental toxins, they contain antioxidant levels 200% higher than those found in fibroblasts [70]. Oxidative stress has been suggested as a key mechanism of sanguinarine action [71], with the greater resistance to sanguinarine cytotoxicity of epidermal keratinocytes (IC_50_ 10 *μ*M) compared to fibroblasts (IC_50_ 1.2 *μ*M) possibly due to the increased antioxidant reserve of these cells.

ZnCl_2_ also has well described cytotoxicity against L929 murine fibroblast cultures, having an IC_50_ of 0.928 *μ*M, classifying it as a highly cytotoxic metal salt [36]. At high concentrations of 80 *μ*g/mL or 0.6 mM, ZnCl_2_ exposure results in 40% HLE B-3 human epithelial cell death by either apoptosis or necrosis [72]. Thus, current in vitro evidence suggests the two main ingredients of black salve, bloodroot and ZnCl_2_, are indiscriminate in their cytotoxic action at concentrations that may be present in black salve preparations.

Black salves contain additional botanical ingredients that may also have normal cell cytotoxicity (Table 1). Chaparral* (Larrea tridentata)* leaves contain multiple phytochemicals including nordihydroguaiaretic acid (NDGA), a polyphenol lignin that constitutes 5–10% of leaf dry weight [73]. HaCaT immortalized human keratinocytes exposed to 150 *μ*M of NDGA undergo apoptosis indicated by a complete disappearance of poly (ADP-ribose) polymerase (PARP) [37]. Some black salves contain 17% NDGA by weight [28]. Graviola* (Annona muricata)* leaves are a rich source of acetogenin (AGEs) long chain fatty acids. Over 100 AGEs have so far been identified in the plant, many with promising antitumour activity [74]. Cytotoxicity has been reported with an* A. muricata* extract against WRL-68 normal hepatic cells, with an IC_50_ of 52.4 *μ*g/mL [75]. Oleander* (Nerium oleander)* may also be a constituent and contains antitumour cardiac glycosides [76] such as oleandrin [77]. Oleandrin at concentrations of 0.6 *μ*g/mL (1.04 *μ*M) did not have a cytotoxic effect on isolated human peripheral blood mononuclear cells or neutrophils [78] but normal cell cytotoxicity has not been assessed at higher concentrations.

As a natural product, the alkaloid composition of* S. canadensis* shows natural variation, being influenced by a number of environmental and genetic factors [79, 80]. Bloodroot rhizomes have been shown to have an up to fifteenfold variation in sanguinarine concentration [81]. When natural products are incorporated into therapeutic agents, it is often difficult to control the concentration and stability of the active ingredients to which patients are exposed. Even if sanguinarine has a narrow window of preferential tumour cell cytotoxicity, with the IC_50_ of A431 epidermoid carcinoma cells being 0.7 *μ*g/mL and normal human epidermal keratinocytes being 3.7 *μ*g/mL [82], small variations in black salve sanguinarine concentration could result in normal cell cytotoxicity. Surprisingly the chemical analysis of black salve has only been reported once in the literature, this being from a single sample with no quantitative analysis performed [47]. Currently the black salve constituent concentration range patients may be exposed to is unknown.

With a number of black salve constituents possessing in vitro normal cell cytotoxicity at low concentrations, health claims regarding black salve tumour specificity appear false. Normal cell cytotoxicity is usually assessed with single agent or single botanical source in vitro assays. Complex herbal formulations such as black salve present a challenge for assessing their cytotoxic potential. Different compounds and botanical extracts may have antagonistic or synergistic effects of relevance to black salve therapeutic efficacy and toxicity.

### 3.1. In Vivo Evidence of Nonselectivity: Histology

Studies that have examined the histopathology of skin following the application of black salve are also counter to claims of a discriminating destructive action. Treated areas typically develop an eschar of dead tissue that sloughs off approximately ten days after salve application, leaving an ulcer [42]. Histologically these ulcers are surrounded by necrosis, suppurative inflammation, and limited residual viable tissue [44]. Moreover, extensive tissue necrosis has been found to develop from the epidermis to the dermis and into subcutaneous tissues [83]. Histological examination of a detached eschar following black salve administration showed it to contain both malignant and normal tissue, contradicting the claim that black salve only damages cancer cells [42].

Black salve may have other pathological implications and has been shown to induce cellular and structural atypia in tissues that can mimic malignancy [83]. In a series of sixteen black salve treated lesions, pathologists needed to perform immunohistochemistry on seven lesions (44%) due to concerning histological features such as atypical spindled cells and necrotic melanocyte containing architecture [44]. Although initially used by patients to treat skin lesions, black salve can result in collateral clinical and histological pseudomalignancies that pose diagnostic challenges.

### 3.2. In Vivo Evidence of Nonselectivity: Mohs Paste

In 1941, Mohs and Guyer, while investigating the reaction of cancerous and normal tissues to various irritants, observed that a 20% solution of ZnCl_2_ chemically killed both benign and malignant tissues while maintaining their microscopic structure [84]. Mohs spent four years developing and testing a fixative paste that would histologically preserve human tissue in a controlled manner. The paste functions like a reservoir that slowly releases ZnCl_2_, the depth of tissue death and fixation determined by the thickness, and surface area of paste application [85].

With standard skin cancer surgery, 1-2% of surgical margins are histologically examined [86]. Mohs believed histological examination of the entire margin for tumour involvement would improve skin cancer cure rates. The Mohs fixed tissue technique involves tumour site fixation with Mohs paste. Horizontal tissue sections are sequentially removed and histologically examined with tumour margin involvement being mapped; further tissue fixative is then applied to persisting tumour until clear margins are obtained [87]. Using the fixed tissue method, Mohs 5-year cure rate, despite a complicated case load where 20% of patients had recurrent disease, was 99.3% for BCC based on 7,257 cases and 94.4% for SCC based on 2,551 cases [88].

In contrast to black salves, where ZnCl_2_ concentration has not been reported, Mohs fixative paste contains 45.2% ZnCl_2_ by weight [84]. Small millimetre-scale changes in paste thickness resulted in significant changes to the depth of fixative penetration. To perform its function, Mohs paste fixes and kills both benign and healthy tissue. The Mohs histology slides of 1000s of patients from the past 75 years attest to the indiscriminate toxicity of salves containing ZnCl_2_ and* S. canadensis*.

The experience of Mohs fixed tissue technique highlights the potentially destructive power of escharotics. Mohs also used his paste to treat gangrene. Applying a thin 2 mm layer to a gangrenous toe for 24 hours fixed the entire toe, facilitating amputation [89]. In this 24-hour timeframe, tissue destruction to a depth of 2 cm can occur [90], with the rapid penetration rate for some normal tissues like cartilage rendering them more susceptible to excessive tissue damage [87].

Black salve has been portrayed as a therapy that selectively destroys tumour cells, enabling it to cure 98–100% of skin cancers with minimal scarring [57]. The indiscriminate in vitro cytotoxicity of both* S. canadensis* alkaloids and zinc chloride, the histological evidence showing normal tissue necrosis in patients that have used black salve, and the adverse event case studies in the medical literature all suggest black salve lacks tumour specificity.

## 4. Is Black Salve Effective at Curing Skin Cancer?

The literature contains 14 journal articles and abstracts that report the use of black salves derived from* S. canadensis* in 19 pathology confirmed skin cancers from 15 individuals (Table 2). While case studies cannot determine efficacy or assess toxicity rates, they can provide information about potential toxicities and characterise treatment failures. Black salves have been used to treat melanoma, squamous cell carcinoma (SCC), and basal cell carcinoma (BCC) with varying results as detailed below.

A number of Internet sites suggest that black salve can be used to treat melanoma effectively [91, 92]. The scientific literature does not support this claim. Of the two documented patients that have used black salve to treat melanoma, both developed metastatic disease [38, 39]. One of these patients had a superficial 0.6 mm Breslow thickness melanoma on her calf with an anticipated 12-year survival rate with conventional surgical management of 92.2% [93].

Nonmelanoma skin cancers have also been treated with black salve. Squamoproliferative lesions include two SCCs [40, 42] and a keratoacanthoma (KA) [41]. While one patient showed no evidence of persisting SCC on partial biopsy of their black salve treatment site, the other two patients showed persisting malignancy. One presented with a 10 cm diameter scalp SCC with perineural invasion and regional lymph node metastases despite black salve application [40].

Basal cell carcinomas (BCCs) have had mixed results when treated by black salve. A total of nine out of 14 BCCs (64%) appeared to be treated successfully. However, only two of these cases had the entire salve treatment area excised and examined histologically [42, 43], with five other cases having a posttreatment biopsy that did not show BCC persistence [44, 48]. Two additional cases were clinically free of recurrence at 6 months' [47] and 12 months' follow-up [49], too short a timeframe to determine if the treatment had been curative.

Black salve BCC treatment failures resulted in 8-stage and 10-stage Mohs surgery being required to completely remove a scalp and separate nose BCC in one patient [45]. Another patient had a nose BCC recur after apparent resolution with black salve. The tumour infiltrated the maxilla requiring partial maxillectomy; the BCC subsequently metastasized to regional lymph nodes with the patient dying of metastatic BCC despite radical neck lymphadenectomy with adjuvant radiotherapy [43].

Conventional surgical skin cancer management has been assessed in multiple clinical trials, individually involving thousands of patients. These have shown primary BCC cure rates of 95.2% for clinical margin surgery [94] and 99.5% for Mohs Micrographic Surgery [95]. Primary SCC has a 5-year recurrence free rate of 94.6% with standard excision [96] and 97.4% with MMS [97]. In melanoma the prognosis varies depending on tumour thickness ranging from a 92% 10-year survival for lesions <1 mm thick to a 50% 10-year survival for lesions >4 mm thick [98]. The majority of lesions are thin melanomas [99]. Despite the likely publication bias favouring the reporting of negative outcomes following black salve use in the medical literature, claims that black salve has superior or equivalent efficacy to surgery are currently not substantiated.

Clinicians are trained to assess the risk that skin lesions pose using variables such as histological subtype, lesion location, diameter, and thickness [100]. Compared to surgical excisions which have a 96% cure rate for BCC [101], the topical cream Imiquimod has a clearance rate for superficial and nodular BCC of 85% and 81%, respectively, [102] but only 56% for infiltrating or aggressive BCC subtypes [103]. For this reason the standard of care for aggressive BCC subtypes is surgical excision [104].

Currently the pharmacokinetics of black salve are not known. While surface eschar formation may be reassuring to patients, the depth of its cytotoxic effect has not been determined. Malignancy may persist under black salve scar tissue and extend subcutaneously before becoming clinically apparent [45]. Without an understanding of skin cancer biology and behaviour, unsuitable lesions can be selected by patients for black salve topical treatment, placing them at increased risk of recurrence and metastatic disease.

## 5. Does Black Salve Cause Less Scarring Than Surgery?

Surgery by primary closure results in healing where wound edges are in apposition. Black salve leaves an open wound that heals by secondary intention. Secondary intention healing begins with basal wound granulation and wound edge contraction followed by subsequent reepithelialization [105]. ZnCl_2_ escharotics have been used to treat venous ulcers [106] and osteomyelitis [107]. In Mohs surgery the fixative paste provides an infection resistant platform for wound healing with rapid epithelialization where defects of 8 cm diameter have healed completely in six weeks [108].

The cosmetic result for healing by secondary intention depends on the site and size of the defect. With facial wounds, concave areas of the nose, eye, ear, and temple (NEET regions) have cosmetic outcomes often superior to transposition flaps and grafts [109, 110]. Wounds on convex areas such as the nasal tip and nasal ala however heal with poor cosmetic results [111]. Smaller wounds <2 cm in diameter predictably heal better than larger wounds, having greater tissue contraction, resulting in less scar tissue deposition [112]. Facial defects involving more than one cosmetic subunit usually have poor cosmetic results with second intention healing [111]. The cosmetic outcome for patients using black salve will depend to some extent on the site, size, and number of cosmetic subunits to which it has been applied. Black salve ulcers are surrounded by inflamed and necrotic tissue [44] from which regeneration tries to occur, the ulcer potentially taking up to 2 years to heal [57].

It is difficult from the available literature to assess definitively the cosmetic outcomes for patients that have used black salve. A standardized measure for cosmesis is not used by authors, with some papers not recording the cosmetic outcome or failing to identify the number of sites where black salve was applied. Within these limitations there are reports relating to a total of 36 patients that have treated 43 skin lesions with black salve. These reports include 19 confirmed malignancies and 24 lesions of either benign histology or where histology was not taken [113, 114]. Of these 43 lesions, four lack a comment on cosmesis, five had a persisting tumour, and nine were in the ulcerative stage. The ulcerative stage follows sloughing of the black salve eschar; as an open wound it prevents an assessment of final cosmesis to be made.

Of the 25 lesions that have some cosmetic outcome information documented (Table 3), the cosmetic outcome for two lesions (8% of black salve assessable cases) is reported as fair to good in the literature. In one of these cases where a nasal tip BCC was treated with black salve for eight consecutive days the patient was left with a depressed, irregular scar which the dermatologist authors described as a fair cosmetic outcome [48]. In the other case, where a R nasal sidewall BCC was treated for 6 days with alternating black and yellow salves at a six-month review the patient had a good cosmetic outcome [47].

In thirteen cases (52%) scarring was noted after black salve application without grading cosmesis. These include a large neck eschar that required management with oral and topical corticosteroids [51], a biopsy proven compound naevus on a 35-year-old woman's thigh that developed into a 5 cm granulomatous plaque [52] and a 29-year-old man who developed cheek scarring that required three scar revision surgeries [42]. Two treatment areas (8%) developed keloid scarring [44] with one requiring cosmetic surgery [43], and an additional three treatment areas (12%) had pigmentation changes necessitating histological assessment [44].

The most graphic cosmetic adverse events occurred in five individuals (20%) that suffered deformity as a consequence of black salve use. All five cases involved the nose with two patients losing an ala unit [43, 46] and another suffering a through-and-through nasal alar defect requiring a hinge flap repair [53]. The most extensive destruction however occurred with two female patients having the majority of their nose destroyed by black salve [54, 55]. These individuals subsequently required a number of reconstructive surgical procedures in an attempt to repair the escharotic damage they sustained.

Although selection bias will favour the reporting of adverse events in relation to black salve, it is notable that, of 25 cases available for cosmetic assessment in the literature, only 8% report a good or fair cosmetic outcome. The number of case studies reporting scarring, keloid formation, and disfiguring deformity suggests black salve has the potential to cause significant cosmetic harm. In contrast, of 174 patients undergoing standard excision, 87% rated their cosmetic result as good [115], while 93% of 228 patients reported a satisfactory or good rating following Mohs surgery [116]. While portrayed as a cosmetically superior treatment to surgery, black salve's mechanism of action and suspected indiscriminate toxicity suggest this is not the case. Black salve has not spared a number of patients from requiring surgery to correct cosmetic damage or treat persisting malignancy.

## 6. Discussion

An increasing number of patients are turning to the Internet to provide them with information about healthcare and treatment options [117]. As an unregulated space, there is the potential for inaccurate or misleading claims to result in choices leading to harmful health outcomes [118].

Mohs was aware of the toxicity of black salve type escharotics, where an extra millimetre of salve thickness could result in significant tissue destruction. For this reason he patented his paste [119] to prevent untrained individuals from using it as a blind chemosurgical technique [120]. Some have suggested that Mohs paste, in addition to facilitating the histological excision of skin cancers, may possess immune enhancing effects [121]. Evidence of improved five-year survival rates in Mohs treated melanoma patients compared to a historical standard excision control cohort furthers these claims [122]. This study however did not determine whether salve pharmaceutical effects or the process of microscopic tumour removal was responsible; it also relied on bias prone historical control data. Claims of an immune adjuvant Mohs paste effect are currently therefore unsubstantiated. The benefit of Mohs paste lies not in its action as a cancer killing therapy but rather in its ability to fix tissue, allowing a cancer to be microscopically mapped and removed. The equivalent cure rates of the Mohs fixed tissue technique utilizing Mohs paste, and the subsequently developed Mohs fresh tissue technique without Mohs paste, attest to this fact [123]. Mohs paste has a very limited role in contemporary clinical practice being reserved for skin tumours that invade bone [124], neoplastic pelvic bleeding [125], and palliative local cancer control [126].

A number of bold claims have been made regarding black salve. Currently the evidence does not exist to confirm or dismiss the claim that black salve has a better cure rate than surgery or that it results in a better cosmetic outcome than surgery for patients. This would require a randomized clinical trial, which has not occurred in the 150-year history of black salve use. Substituting highly effective conventional therapies with an unproven alternative such as black salve should not occur outside the framework of a clinical trial.

Regulators have been trying to protect consumers from black salve by policing the claims vendors post on their websites [127] and by making it illegal to import or sell black salve [128]. A number of loopholes however exist in the regulatory framework. Vendors may relocate their operations to countries with reduced regulatory controls [57], black salve ingredients can be legally imported from which patients can manufacture their own salve [129], and black salve veterinary products can be imported and used by humans [130]. While regulation is a valuable pillar in harm prevention, it is failing to limit escharotic salve use to treat skin cancer.

Skin cancer is the most common malignancy in the western world. The annual cost to the US health system from 2007 to 2011 was $8.1 billion, with skin cancer expenditure growing 126.2% over the 5-year period compared to a 25.1% cost increase for all other cancers [131]. In 2007 $34 billion was spent on CAM in the US [6]. With growing demand and an absence of clinical evidence assessing the benefits and risks of black salve, patients will continue experimenting. Observational studies and clinical trials offer a mechanism for assessing the efficacy of black salve; they may also facilitate harm minimisation by discouraging black salve use for melanomas, high risk nonmelanoma skin cancers, and obviously benign lesions.

## 7. Conclusion

Black salve is not a natural therapy. It contains significant concentrations of synthetic chemicals. Black salve does not appear to possess tumour specificity with in vitro and in vivo evidence indicating normal cell toxicity. Black salve does appear to cure some skin cancers, although the cure rate for this therapy is currently unknown. The use of black salve should be restricted to clinical research in low risk malignancies located at low risk sites until a better understanding of its efficacy and toxicity is developed. Where a therapy capable of harm is already being used by patients, it is ethically irresponsible not to study and analyse its effects. Although cautionary tales are valuable, black salve research needs to move beyond the case study and into the carefully designed clinical trial arena. Only then can patients be properly informed of its true benefits and hazards.

## Figures and Tables

**Table 1:** Black salve constituent normal cell cytotoxicity.

Compound	Cell type	IC50 (*μ*M)	Ref
Sanguinarine	Skin fibroblasts KF-II	1	[32]
	Gingival fibroblasts	1.2	[33]
	Epidermal keratinocytes	10	[34]

Chelerythrine	Skin fibroblasts KF-II	1.5	[32]
	Gingival fibroblasts	4.7	[33]

Sanguilutine	Lung fibroblasts	1.2	[35]

Chelilutine	Lung fibroblasts	1.7	[35]

Sanguirubine	Skin fibroblasts KF-II	0.53	[32]

Chelirubine	Skin fibroblast KF-II	0.5	[32]

Zinc chloride	Murine fibroblast	0.93	[36]

NDGA	HaCaT keratinocytes	150	[37]

**Table 2:** Black salve skin cancer treatment clinical outcomes.

Cancer type	Number of cases	Outcome	Ref
Melanoma	2	2 metastatic melanomas	[38, 39]

SCC	3	1 metastatic SCC	[40]
		1 persisting SCC	[41]
		1 recurrence-free histologically	[42]

BCC	14	1 metastatic BCC	[43]
		4 persisting BCC	[44–46]
		3 recurrence-free clinically	[47–49]
		6 recurrence-free histologically	[42–44]

**Table 3:** Black salve cosmetic results.

Cosmetic outcome	Number of cases	Ref
Fair to good	2	[47, 50]
Scarring	13	[42, 51, 52]
Keloid scarring	2	[43, 44]
Concerning pigmentation changes	3	[44]
Deformity	5	[43, 46, 53–55]
